# Feasibility and Surgical Outcomes of Hysteroscopic Myomectomy of FIGO Type 3 Myoma: A Systematic Review

**DOI:** 10.3390/jcm12154953

**Published:** 2023-07-27

**Authors:** Andrea Etrusco, Antonio Simone Laganà, Vito Chiantera, Amerigo Vitagliano, Ettore Cicinelli, Mislav Mikuš, Marina Šprem Goldštajn, Federico Ferrari, Stefano Uccella, Simone Garzon, Sandro Gerli, Alessandro Favilli

**Affiliations:** 1 Unit of Gynecologic Oncology, ARNAS “Civico—Di Cristina—Benfratelli”, Department of Health Promotion, Mother and Child Care, Internal Medicine and Medical Specialties (PROMISE), University of Palermo, 90133 Palermo, Italy; etruscoandrea@gmail.com (A.E.); antoniosimone.lagana@unipa.it (A.S.L.); vito.chiantera@unipa.it (V.C.); 2 1st Unit of Obstetrics and Gynecology, Department of Biomedical and Human Oncological Science (DIMO), University of Bari, 70121 Bari, Italy; amerigo.vitagliano@gmail.it (A.V.); ettore.cicinelli@uniba.it (E.C.); 3 Clinical Hospital Center Zagreb, Department of Obstetrics and Gynecology, 1000 Zagreb, Croatia; marina.goldstajn@gmail.com; 4 Department of Clinical and Experimental Sciences, University of Brescia, 25136 Brescia, Italy; federico.ferrari@unibs.it; 5 Department of Obstetrics and Gynecology, AOUI Verona, University of Verona, 37129 Verona, Italy; stefano.uccella@univr.it (S.U.); simone.garzon@univr.it (S.G.); 6 Section of Obstetrics and Gynecology, Department of Medicine and Surgery, University of Perugia, 06123 Perugia, Italy; sandro.gerli@unipg.it (S.G.); alessandro.favilli@unipg.it (A.F.)

**Keywords:** myoma type 3, uterine fibroids, hysteroscopy, myomectomy, female infertility

## Abstract

**Background**: The latest classification from the Fédération Internationale de Gynécologie et d’Obstétrique (FIGO) has reclassified type 3 myomas, changing their classification from intramural to submucosal. While hysteroscopic myomectomy is considered the gold standard treatment for patients experiencing symptoms from submucosal myomas, there are currently no specific guidelines available for managing type 3 myomas, and the optimal surgical approach remains uncertain. **Methods**: The search for suitable articles published in English was carried out using the following databases (PROSPERO ID CRD42023418602): MEDLINE, EMBASE, Global Health, The Cochrane Library (Cochrane Database of Systematic Reviews, Cochrane Central Register of Controlled Trials, Cochrane Methodology Register), Health Technology Assessment Database, Web of Science and search register. Only original studies reporting data on hysteroscopic myomectomy of type 3 myoma were considered eligible. The main outcomes investigated were the effectiveness and feasibility of hysteroscopic myomectomy and reproductive outcomes after surgical treatment. **Results**: Two hundred and sixty-one studies were screened and nineteen of these were read for eligibility. Three studies encompassing 56 patients in total were included. Among the overall population studied, three patients needed an additional procedure to completely remove the myoma and five cases of post-surgical synechiae were recorded. No complications were reported. Of 42 patients wishing for pregnancy, the cumulative live birth rates before and after the hysteroscopic myomectomy were 14.3% and 42.9%, respectively. **Conclusions**: Hysteroscopic myomectomy appears to be a safe and feasible approach. Nevertheless, data reported in the literature are extremely scarce and based on studies with few patients enrolled. New evidence is needed to assess the safety and effectiveness of hysteroscopic treatment for FIGO type 3 myomas.

## 1. Introduction

Uterine fibroids are benign monoclonal tumors of the smooth muscle cells of the myometrium [1] and represent the most common pathology in the female genital tract [2]. The incidence ranges from 5.4% to 77%, and it is affected by factors such as ethnicity, age and the diagnostic method used, making it challenging to provide an accurate estimate [3]. Although most myomas are asymptomatic, some, depending on their location, size and number, can be responsible for pelvic pain, abnormal uterine bleeding (AUB) and states of subfertility and infertility [4].

Despite countless numbers of papers available in the literature in this field, paradoxically there are several issues regarding the effects and management of uterine myomas which are still to be clarified. In order to obtain robust evidence in this regard, in 2011, the Federation Internationale de Gynecologie et d’Obstetrique (FIGO) released a new system for the classification of uterine fibroids [5] with the aim of providing a new consistent and universally accepted nomenclature [6]. The new subclassification system was articulated in eight different classes (types) of myomas according to their position in the uterus, allowing one to overcome and improve upon the previous old and gross nomenclature: subserosal (SS), intramural (IM) and submucosal myomas (SM). 

Since the last update in 2018, the classification of type 3 uterine fibroids has changed. These fibroids, which have myometrial development but encroach upon the endometrium, are now included as submucous leiomyomas. They are distinguished from type 2 fibroids through hysteroscopy, using the lowest possible intrauterine pressure necessary to allow visualization [6].

Despite the fact that hysteroscopic myomectomy is the gold standard treatment for patients affected by SM myomas complaining about AUB and/or with an infertility or subfertility history [7,8,9,10,11], for type 3 myomas management there are no guidelines available, and the best surgical approach is not yet clarified.

Taking into account that the type 3 myomas could be considered as submucous ones, we performed a systematic review of the literature with the aim of evaluating the feasibility and the surgical outcomes of hysteroscopic treatment.

## 2. Materials and Methods

A systematic review was conducted through a search on the following databases: MEDLINE, EMBASE, Global Health, The Cochrane Library (Cochrane Database of Systematic Reviews, Cochrane Central Register of Controlled Trials, Cochrane Methodology Register), Health Technology Assessment Database and Web of Science research registers. The systematic review was registered in PROSPERO (ID: CRD42023418602) before starting the search and followed the Preferred Reporting Items for Systematic Reviews and Meta-Analyses (PRISMA) guideline [12], validated by the Enhancing the Quality and Transparency of Health Research (EQUATOR) network, and the Cochrane Handbook for Systematic Reviews [13].

We used the medical subject heading (MeSH) term “Leiomyoma” (MeSH Unique ID: D007889) in combination with “Hysteroscopy” (MeSH Unique ID: D015907) and “Uterine Myomectomy” (MeSH Unique ID: D063186) and “Myoma” (MeSH Unique ID: D009214), and “Type 3”. We selected papers written in English, since the inception of each database until 30 April 2023.

Titles and/or abstracts of studies retrieved using the search strategy, and those from additional sources, were screened independently by 2 review authors (A.E., A.F.) to identify studies that potentially met the aims of the systematic review. The full texts of these potentially eligible articles were retrieved and independently assessed for eligibility by 2 other review team members (A.S.L., A.V.). Any disagreement between them over the eligibility of articles was resolved through discussion with a third (external) collaborator. We selected only cohort (retrospective and prospective), clinical or case-control studies, and case reports or case series reporting hysteroscopic myomectomy of type 3 myoma. We excluded studies encompassing type 3 myomas with aggregated results with other myoma types and/or with no mention of outcomes by subtype.

Two authors (A.E., A.F.) independently extracted data from articles about study characteristics and included populations, methods and results/outcomes, using a pre-piloted standard form in order to ensure consistency. Any discrepancies were identified and resolved through discussion (with a third external collaborator where necessary). Due to the nature of the findings, we opted for a narrative synthesis of the results.

## 3. Results

### 3.1. Study Selection

**Figure 1:**
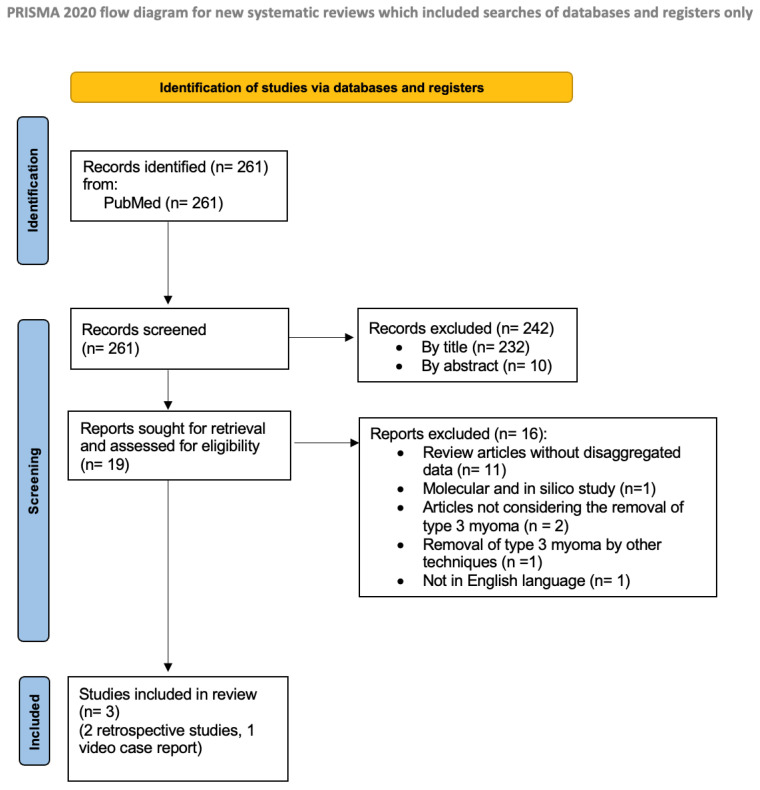
Preferred reporting items for systematic reviews and meta-analyses (PRISMA) flow diagram.

The literature search based on our pre-defined key search items identified 261 publications, after removing duplicates. The title and abstract of manuscripts were screened, resulting in 19 studies considered potentially eligible to be included in the review. After the evaluation of the full text, 16 studies were excluded: 11 manuscripts [14,15,16,17,18,19,20,21,22,23,24] were articles without disaggregated data; 1 molecular and in silico study [25]; 2 articles not considering the removal of type 3 myoma [26,27]; 1 study where type 3 myoma removal was performed by other techniques [28]; 1 additional study was not in the English language [29]. Finally, a total number of three studies [30,31,32] that met the abovementioned inclusion criteria were included in the present systematic review (Figure 1).

As summarized in Table 1, the studies embedded a total of 56 patients who underwent hysteroscopic myomectomy for type 3 myomas; 2 were retrospective studies [30,31] and 1 was a video case report [32] coming from France, China and the United Kingdom, respectively. All studies were published in English.

### 3.2. Analysis of the Reports

In two articles, the main outcome was to assess the effectiveness and feasibility of hysteroscopic myomectomy [30,32], whereas one retrospective case-control study aimed to evaluate surgical outcomes and the effect of hysteroscopic resection of type 3 fibroids on the pregnancy outcomes in infertile women [31]. The baseline characteristics of the patients included are listed in Table 2.

In chronological order, the first study was a retrospective analysis conducted by Capmas et al. [30] on 13 women affected by type 3 myoma who underwent hysteroscopic myomectomy. Among these patients, ten were suffering from AUB, two from infertility and one from pelvic pain. The mean size of the resected myomas was 3.08 cm and 31% of patients presented multiple myomas. The surgery was performed by two experienced surgeons. The procedure started with the incision of the endometrium with a twizzle electrode by a Bettocchi hysteroscope and then by a 26 Fr resectoscope with a Collins loop. Successively, myomas were resected by classical slicing. For three patients, it was not possible to obtain a total resection in a single surgical time, and for this reason they had to undergo a second operative hysteroscopy. An additional procedure was required in four out of eight women wishing for pregnancy in order to obtain a normal uterine cavity. In three patients (23%), the presence of synechiae was found at the diagnostic hysteroscopy follow-up (two cases of type I and one case of type II according to March classification [33]) and required hysteroscopic adhesiolysis. No post-operative complications were reported. Bleeding control was obtained in seven women out of nine. The study did not mention the fertility outcomes of the two patients who wished for pregnancy.

The second study included was a video case report [32] illustrating the technique to be used to perform a hysteroscopic myomectomy in a 35-year-old patient with a history of primary infertility affected by a 3 cm type 3 myoma of the posterior uterine wall. Hysteroscopic surgery was performed according to the classic slicing technique with pseudocapsule sparing. No post-operative complications were recorded. The patient underwent a diagnostic hysteroscopy follow-up 8 weeks after the surgery in which an intact endometrium was found. The woman then underwent in vitro fertilization (IVF) successfully.

The last study included was a retrospective case-control study conducted by Han et al. [31] with the aim of evaluating the effect of type 3 myomas on IVF cycle outcomes and whether these were modified by hysteroscopic myomectomy. In total, 101 patients with type 3 fibroid were divided into two groups: 59 non-surgical (among them, 5 had a combination of SSs with type 3 myoma and 2 a combination of SSs with multiple type 3 myomas) and 42 surgical (6 suffering from multiple type 3 myomas). These were matched to a control group of 61 patients with a normal uterus (1:1 match ratio). The myomectomy was performed by a single experienced surgeon (>10 years of experience and >500 achieved operative hysteroscopies per year) using a 26 Fr bipolar hysteroscope equipped with a 30-degree lens. In order to facilitate the myoma dislocation toward the uterine cavity, distension media pressure was gradually reduced and an intravenous infusion of 10 UI of oxytocin in 500 mL of saline solution (0.9%) at a rate of 120 mL/h was administrated during the procedure. No complications were recorded. The mean size of the resected myomas was 2.45 cm and six patients were treated for multiple myomas. All procedures were performed under ultrasound control. No residual fibroids, abnormal uterine bleeding or infection were reported at the ultrasound and diagnostic hysteroscopy follow-up performed 6–8 weeks after the surgery. Mild intrauterine adhesions were diagnosed in two patients who needed hysteroscopic adhesiolysis. Regarding the reproductive outcomes, no significant differences in terms of cumulative clinical pregnancy rate and cumulative live birth rate were reported between the control and surgery groups.

## 4. Discussion

The FIGO subclassification system for uterine myomas has allowed us to overcome the limits of the old classification, which has proved to be inadequate to obtain solid evidence, and probably contributed (at least in part) to shedding light on the grey area regarding the effects and management of uterine fibroids. Nevertheless, the novelties introduced by such a new classification have offered a new point of view for clinicians and researchers, but also new clinical dilemmas.

For a long time, before the advent of the FIGO subclassification system, type 3 myomas were considered as IM ones, and therefore the effects exerted in terms of AUB and fertility have been lost and generalized among fibroids lying within the uterine wall.

Recent findings suggest that type 3 myomas may negatively impact fertility, raising questions about the effectiveness of treatment options and the best approach to addressing these lesions [34]. A pharmacological treatment would allow for the avoidance of treatment causing undesirable scars to the uterus, but no solid evidence is available in this regard [35,36]. Unfortunately, robust evidence and guidelines are still lacking about surgical treatments as well.

During pregnancy, especially in the first trimester and early second trimester, fibroids tend to grow extensively [37]. Due to this extensive growth, the fibroid may excessively increase the blood supply, leading to inadequate oxygenation and, consequently, necrosis. There is evidence that inflammation triggered by fibroid necrosis can increase the risk of pre-term delivery [38]. For type 3 myomas and SMs in general, given their extreme proximity to the uterine cavity, the risk of pre-term delivery may be further increased. Management of these types of myomas should therefore be considered in women with a history of subfertility and/or pre-term delivery and repeated pregnancy losses.

The main principle to be followed when performing a myomectomy is to save as much myometrial tissue as possible. Based on the anatomical features of type 3 myomas and the surgical evidence available regarding the treatment of uterine fibroids, to date it can be assumed that the best way to perform a myomectomy for a type 3 myoma may be hysteroscopy. The proximity to the endometrium allows the saving of more myometrial fibers than other known surgical approaches. Furthermore, hysteroscopic myomectomy, if performed respecting the pseudocapsule, can take advantage of the anatomical characteristics of the fibroid itself. The surgical action of bluntly disconnecting the fibroconnective bridges that anchor the myoma to the pseudocapsule guarantees the myometrial sparing treatment [39]. Moreover, the importance of the pseudocapsule has been emphasized as a natural limitation to the surgeon’s action, but also for its fundamental role in the healing of the myometrium after myomectomy. In this regard, the hysteroscopic cold loop myomectomy has proven to be a safe and effective technique that allows the myoma to be enucleated in a single surgical procedure with a low risk of post-surgical synechiae [40].

Nevertheless, currently there are no clear guidelines on how to treat patients with FIGO type 3 myoma. In view of pieces of evidence that have emerged from this systematic review, knowledge about the hysteroscopic treatment of this kind of myoma is poor and mainly aimed at investigating the effectiveness and feasibility in patients wishing for pregnancy. This issue could be explained by the available alternative treatments for patients complaining of AUB, such as an intrauterine device containing progesterone.

In this regard, the study by Capmas et al. [30] was the first to demonstrate that hysteroscopic myomectomy, when performed by an experienced surgeon, is feasible for type 3 myoma. However, the patient populations in these studies were extremely small, many patients underwent more than one procedure and three out of eight patients (37.5%) experienced post-surgical synechiae. This is a significant concern, as for a patient hoping to become pregnant, developing intrauterine synechiae after hysteroscopic myomectomy could lead to replacing one problem with a more complicated one [41]. It could be speculated that the classical slicing technique, even performed by an experienced surgeon, did not allow them to ensure a myoma resection without myometrium injury. This hypothesis seems to be supported by the fact that no multiple myomas were treated in this series, which could have increased the risk of post-surgical synechiae. Nevertheless, Vorona et al. [32] reported the case of only one patient successfully treated by a classical slicing technique obtaining a recovered uterine cavity at the post-surgical diagnostic hysteroscopy follow-up.

Han et al. [31] were the only ones to have evaluated reproductive outcomes before and after the hysteroscopic myomectomy. Also in this study, hysteroscopic myomectomies were performed by an expert surgeon by classical slicing under an ultrasound guide. Differently from other reports, after surgery, all patients received oral continuous combined 2 mg 17-β estradiol and 10 mg dydrogesterone for two menstrual cycles to promote the recovery of the endometrium. Only two patients reported post-surgical synechiae, subsequently removed by operative hysteroscopy. Nevertheless, the hysteroscopic myomectomy of type 3 myomas did not significantly improve reproductive outcomes in terms of cumulative pregnancy rate and cumulative live birth rate. The statistical power of these findings, however, is extremely limited by the low number of patients involved in the study.

The main limitation of this systematic review is represented by the small number of papers included in our analysis, which resulted in a limited number of patients being studied. We believe that this scarcity of evidence could be partially explained by the recent inclusion of type 3 fibroids as ‘submucous’ in the FIGO subclassification system, which only occurred in the last update in 2018. This new classification opened up the possibility of hysteroscopic treatment for these fibroids, but it may take time to accumulate a substantial body of evidence on their hysteroscopic management. Additionally, despite the FIGO subclassification system for uterine fibroids being initially published in 2011, it has not yet gained widespread adoption worldwide [42]. Despite the objective limitations mentioned above, several strengths should be acknowledged. Firstly, the construction of the search strategy demonstrates methodological rigor, as it encompasses a comprehensive search of the most important databases available. The search protocol was constructed according to best practice guidelines, and its details have been assessed, registered and made available online. The objective and search methods are clearly defined, ensuring transparency and reproducibility. As a result of the diligent search strategy, the papers included in this review currently constitute the only available evidence on the hysteroscopic treatment of FIGO type 3 myomas, which can contribute to the understanding of hysteroscopic treatment for this specific type of myoma. Importantly, to the best of our knowledge, this appears to be the first systematic review specifically focused on the hysteroscopic treatment of FIGO type 3 myomas.

## 5. Conclusions

To the best of our knowledge, this is the first systematic review on hysteroscopic myomectomy for type 3 myomas.

The absence of established guidelines on the treatment of type 3 myoma leaves a challenging dilemma about the best approach with which to treat this population. This gap gains more importance considering the detrimental effect that type 3 myomas could exert in terms of fertility.

To date, despite the fact that hysteroscopic myomectomy appears to be a safe and feasible approach, data reported in the literature are extremely poor and based on studies with few patients enrolled. In light of these findings, this treatment should be confined to experienced surgeons, as surgical technical skills are needed to adequately perform the procedure and avoid potential complications.

Further studies should focus on verifying the safety and effectiveness of hysteroscopic myomectomy for type 3 myomas, determining the optimal technique to use and exploring whether reproductive outcomes can be improved for patients who undergo this procedure.

## Figures and Tables

**Table 1:** Characteristics of the included studies.

Author	Year	Type	Main Outcome	Country	Patient (n)	Age (Mean)	Control Group
**Capmas et al. [30]**	2016	Retrospective cohort study	Hysteroscopic myomectomy outcome and feasibility	France	13	42.62	None
**Han et al. [31]**	2022	Retrospective case-control study	Average transfer time to live birth, clinical pregnancy rate, cumulative live birth rate, hysteroscopic myomectomy outcome.	China	42	33.41 ± 4.24	61 controls (normal uterus) 59 non-surgery (with FIGO type 3 myomas)
**Vorona et al. [32]**	2022	Case report	Hysteroscopic myomectomy outcome and feasibility	UK	1	35	None

**Table 2:** Baseline characteristics and hysteroscopic myomectomy outcomes of the included studies.

	Campas et al. [30]	Han et al. [31]	Vorona et al. [32]
**Characteristics of the surgeons**			
Surgeon (n)	2	1	1
Years of experience (n)	>2	>10	\
Operative HSC ^1^ achieved per year (n)	>100	>500	\
**Patients (n)**	13	42	1
**Mean age (years)**	42.62	33.41 ± 4.24	35
**Symptoms**			
Irregular bleeding (n)	10	0	1
Infertility (n)	2	42	1
Pain (n)	1	0	0
Symptoms’ durations (y)	\	4.21 ± 2.55	\
**Characteristics of myoma**			
Size of myoma (mean)	3.08 cm	2.45 cm	3
More than 4 cm	31%	0	0
Multiple myoma (n)	0	3	0
**Surgery items**			
Ultrasound guided procedures (n)	3	42	0
Mean operative time (min)	50.38	\	\
Post-operative complications (n)	0	0	0
Need for two surgeries (n)	4	0	0
**Surgery outcomes**			
Irregular bleeding after first surgery (n)	10	0	0
Pain after first surgery (n)	1	0	0
Live birth rate before surgery	\	14.3%	0%
Live birth rate after surgery	\	42.9%	100%
Clinical pregnancy rate before surgery	\	28.6%	0%
Clinical pregnancy rate after surgery	\	42.9%	100%
Incomplete resection (n)	3	0	0
Complications	Synechiae (n = 3)	Synechiae (n = 2)	0
Recurrences (n)	3	0	0
**Post-operative hysteroscopy**	Recommended for all the participants	Recommended for all the participants	Recommended
**Post-operative hysteroscopy (n)**	8	42	1
**Additional procedures (n)**	4	2	0
**Months of follow-up (mean)**	48	18	\

^1^ HSC: hysteroscopy.

## Data Availability

Not applicable (no new data were generated during the development of this systematic review).
